# Advances in the mechanism and therapies of achondroplasia

**DOI:** 10.1016/j.gendis.2024.101436

**Published:** 2024-09-24

**Authors:** Hangang Chen, Ruobin Zhang, Min Jin, Jing Yang, Lin Chen, Yangli Xie

**Affiliations:** a Department of Wound Repair and Rehabilitation Medicine, State Key Laboratory of Trauma and Chemical Poisoning, Trauma Center, Research Institute of Surgery, Daping Hospital, Army Medical University, Chongqing 400042, China; b Department of Orthopedic Surgery, The Second Affiliated Hospital, Chongqing Medical University, Chongqing 400010, China

**Keywords:** Achondroplasia, FGFR3, Mechanisms, Skeleton development, Therapeutic interventions

## Abstract

Achondroplasia (ACH), is the prevailing type of genetic dwarfism in humans, caused by mutations in fibroblast growth factor receptor 3 (FGFR3) that are inherited in an autosomal dominant manner. FGFR3 is mainly expressed in condensed mesenchyme, chondrocytes, and mature osteoblasts and osteoclasts, in which it regulates the formation, development, growth, and remodeling of the skeletal system. Mutations in FGFR3 causing ACH result in enhanced FGFR3 signaling through combined mechanisms including enhancing FGF dimerization and tyrosine kinase activity and stabilizing FGF receptors. In ACH, suppression of the proliferation and maturation of chondrocytes in the growth plate leads to a notable reduction in growth plate size, trabecular bone volume, and bone elongation through a profound enhancement of FGFR3 signaling. This review aims to comprehensively outline the cellular and molecular mechanisms contributing to the pathological process of ACH and its potential therapeutic interventions.

## Introduction

Achondroplasia (ACH), the most common form of genetic skeletal dysplasia, affects approximately 1 in every 25,000–30,000 individuals.^1^ Sporadic mutations account for 80% of ACH cases. Due to its distinct chondrodysplasia phenotype, ACH is typically diagnosed at birth.^2^

**Figure 1:**
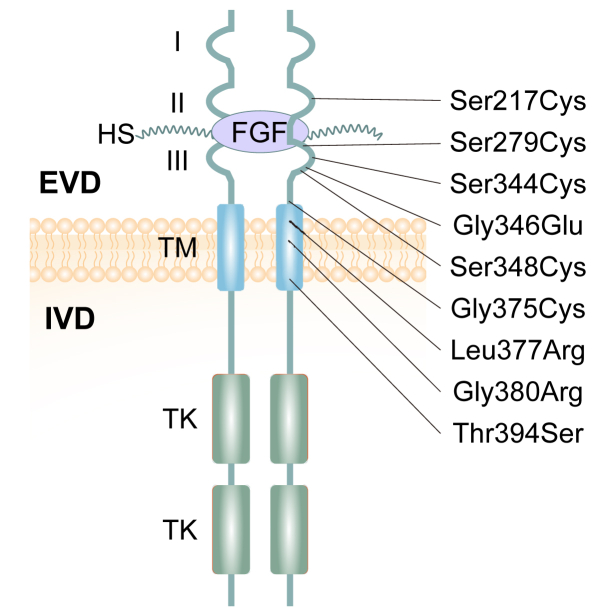
The mutational spectrum of fibroblast growth factor receptor 3 (FGFR3) in individuals with achondroplasia. In humans, the mutations associated with achondroplasia patients are mainly located in transmembrane (TM) domain of FGFR3. The extracellular domain is referred to as ECD, while the intracellular domain is referred to as ICD. Heparan sulfate is represented by HS, and immunoglobulin-like domains are represented by I, II, and III. Tyrosine kinase domains are represented by TK.^5^

ACH is an autosomal dominant inherited disease and its gene locus was first mapped to fibroblast growth factor receptor 3 (FGFR3) by linkage studies using DNA markers in 1994.^3^ Over 97% of ACH cases are caused by a missense mutation (p.Gly380Arg) of FGFR3 in the transmembrane domain.^4^ In comparison to wild-type FGFR3, this mutant exhibits a higher tendency for activation without requiring FGF ligands. ACH may occur with relatively less frequency due to other FGFR3 mutations such as p. Ser217Cys, p. Ser279Cys, and p. Ser344Cys. The presence of these genetic changes in FGFR3 introduces an additional cysteine residue, subsequently leading to the continuous activation of FGFR3 (as shown in Fig. 1).^5^ FGFR3 signaling may also be enhanced by decreased lysosomal degradation of the mutant FGFR3.^6^

Individuals with ACH display a disproportion in stature due to the rhizomelic diminishment of their extremities. ACH patients not only exhibit common facial features such as frontal bossing and hypoplastic midfaces, but also display lumbar lordosis, genu varum, and trident hands.^3^ The progressive disorganization of growth plates and stunted growth of ACH patients suggest that FGFR3 plays a crucial role in controlling the process of endochondral ossification.^7^ In certain individuals with ACH, there may be a partial premature fusion of coronal and sagittal sutures, suggesting the pivotal role of FGFR3 in the regulation of intramembranous ossification.^3^^,^^8^ A study has shown that ACH patients exhibit delayed bone age, a phenomenon that becomes less noticeable during adolescence because of accelerated bone maturation (catch-up growth).^9^ Individuals with ACH experience a notable curvature of the upper spine, resulting in deteriorating deformities as time progresses.^10^ Furthermore, ACH individuals exhibit an exaggerated curvature of the lower back as they grow older. The narrowing of the spinal canal, which is a severe complication of ACH, is caused by both congenital dysplasia and acquired degenerative changes.^11^

We in this review comprehensively outline the cellular and molecular mechanisms that contribute to the pathological process of ACH and its potential therapeutic interventions.

## Processes of skeletal development

Mammalian skeleton in different parts is derived from distinct cell lineage. Neural crest cells give rise to dentin in teeth, as well as specific bones and cartilages located in the face and anterior part of the skull. Mesodermal cells originating from the prechordal region give rise to cartilage and bone tissue, forming the posterior part of the skull. The paraxial mesoderm (somites) forms the axial skeleton, while the appendicular skeleton originates from the lateral plate mesodermal cells.^12^

Skeleton formation initiates when mesenchymal cells originating from the aforementioned embryonic lineages migrate to the locations where the bones will form in the future. Afterward, these cells experience condensation, leading to a higher density of cells that determine the anticipated shape and size of the forthcoming bones. Within these condensations, mesenchymal cells undergo distinct differentiation, either changing into chondrocytes to form anlagen for future bones through endochondral ossification or transforming into osteoblasts to directly produce bone via intramembranous ossification.^12^ Endochondral bone formation is a prevalent process observed in most bones, including the vertebrae of the axial skeleton, the limbs of the appendicular skeleton, and the base of the skull. Conversely, the membranous neuro- and viscero-cranium, along with the inner portion of the clavicle, undergo intramembranous bone formation.

The formation of bones through endochondral ossification is an intricated and synchronized process with multiple steps, which involves mesenchymal cell condensation, the generation of cartilage precursor through chondrogenic differentiation, the arrangement of chondrocytes within the growth plate, the development of hypertrophic chondrocytes, and finally the substitution of cartilage with bone via osteogenesis.^13^, ^14^, ^15^, ^16^ The growth plate serves as the central site for endochondral ossification, facilitating a continuous expansion of chondrocytes.^17^ In terms of morphology and function, the growth plate is divided into four distinct zones, namely resting zone, proliferative zone, pre-hypertrophic zone, and hypertrophic zone.^18^ Adjacent to the surface of the joint, the zone referred to as the resting zone is made up of small circular chondrocytes and established as a niche of stem/progenitor cells. These cells differentiate into flattened chondrocytes, creating the proliferating zone. Chondrocytes secrete aggrecan (ACAN), type II collagen (Col2), and other matrix proteins during their resting and proliferating phases. Subsequently, the chondrocytes situated in the upper part of the hypertrophic zone experience a growth in their dimensions, whereas those in the lower part undergo the process of matrix mineralization. This process is identified by the presence of proteins like type X collagen and matrix metalloproteinase 13 (MMP13). Finally, the replacement of cartilage with bone tissue occurs through a complex process involving the absorption of calcified cartilage, the invasion of blood vessels that bring osteoblast-producing mesenchyme, and osteoclast-producing progenitors, resulting in the formation of bone tissue *in situ* following the programmed cell death of terminal hypertrophic chondrocytes.^19^ Several studies have indicated that hypertrophic chondrocytes possess the ability to transform into osteoblasts, thereby playing a direct role in the formation of trabeculae.^20^^,^^21^ During skeletal development, chondrocytes are reserved to form a dynamic growth plate, which serves as the main structure responsible for the growth of skeletal elements longitudinally.^15^ Recent studies indicate that chondrocytes in the resting zone expressing parathyroid hormone-related protein (PTHrP) may serve as skeletal stem cells, while cells positive for Axin2 and responsive to Wnt signaling in the perichondrial groove of Ranvier, located adjacent to the resting zone, display characteristics of chondroprogenitor cells, contributing to the growth of the growth plate laterally in mice.^22^^,^^23^ In humans, the growth plates gradually diminish in thickness and ultimately ossify, leading to the cessation of longitudinal bone growth in adulthood.

Synovial joints consist of multiple tissue elements, such as articular cartilage that envelops the bone surface, meniscus, synovium that protects the joint from the external environment, and intra-joint and peri-joint ligaments that provide mechanical stability. Articular cartilage can be divided into distinct zones histologically and phenotypically.^24^ Within the superficial zone, cells assume an elongated and flat morphology, aligning in parallel with the articular surface and mainly surrounded by type I collagen. The superficial zone harbors precursor cells of articular cartilage and generates lubricants.^25^ The chondrocytes in the middle zone are round-shaped and organized in vertical column-like stacks. They produce and maintain the essential extracellular proteins, particularly Col2 and ACAN indispensable for resilience. Tidemark is the interface between calcified and non-calcified cartilage. Studies with mammalian embryos indicate that the histological indication of joint formation is the appearance of a dense non-vascularized layer of mesenchymal tissue at the prospective joint locations. The area is called the “interzone” because it disrupts the neighboring cartilaginous components. The interzone is comprised of three distinct layers, namely, two outer layers located adjacent to the epiphyseal end of the developing long bone and the middle layer referred to as the intermediate zone. The outer layers are chondrogenic and express Col2, while the intermediate zone exhibits a non-chondrogenic phenotype, which is identified by the lack of genes specific to chondrocytes such as SRY-box transcription factor 9 (*Sox9*) and *Col2*, and the presence of a set of genes including growth and differentiation factor 5 (*Gdf5*), *Wnt4*, and *Wnt9a* then give rise to articular chondrocytes. We currently still do not fully understand the mechanisms that regulate the expression of these genes in the intermediate zone.^26^ Additional differentiation processes and mechanisms such as the movement of muscle participate in the cavity formation and the development of other joint tissues including meniscus and ligaments.^27^ Recent research has discovered that cells expressing Gdf5 and FGFR2 play pivotal roles in the formation of various tissues, such as the meniscus, cruciate ligaments, and articular cartilage, within synovial joints.^28^

The axial skeleton and its relevant skeletal muscles originate from the somites.^29^ After receiving various patterning signals from neighboring tissues, somites go through differentiation, subsequently leading to the organization of multipotent somitic cells into epithelial dorsal dermomyotomes and mesenchymal ventral sclerotomes. The sclerotomal cells surround the notochord, thereby giving rise to the vertebral bodies, intervertebral discs (except for the nucleus pulposus), and ligaments of the vertebral column. Multiple essential signals, such as homeobox (HOX), retinoic acid (RA), Wnt, and FGF signaling, are necessary for the development of somites.^29^

Skeleton is not a dormant entity. Instead, following the completion of growth, it undergoes constant remodeling to adapt its structure and mechanical characteristics in response to environmental stimuli. Bone remodeling involves simultaneous processes of both bone formation and resorption that are regulated by both systemic and local microenvironments, encompassing a range of molecules.^30^^,^^31^

## Pathophysiology of ACH

ACH patients show short stature (limbs and spine), spinal deformity, and cranial dysplasia. The expression of FGFR3 is primarily detected in chondrocytes situated at the center of the condensed mesenchyme and in all chondrocytes of growth plates except hypertrophic chondrocytes.^32^, ^33^, ^34^, ^35^, ^36^ It is also found in osteoblasts and osteocytes in long bones, as well as in periosteal and sutural osteogenic fronts of the skull.^37^^,^^38^ ACH patients exhibit disrupted bone development and homeostasis, as evidenced by the phenotypes and spatiotemporal expression patterns of FGFR3.

### FGFs/FGFR3 signaling in endochondral bone growth

Mice carrying the FGFR3 mutation that leads to ACH display shortened long bones with disarranged chondrocytes in growth plates and spinal stenosis, as well as cranial deformities characterized by dome-shaped skulls, which closely resemble the phenotypes of ACH patients,^39^^,^^40^ indicating the crucial role of FGFR3 in regulating of endochondral bone formation.

Through the utilization of cultured chondrocytes, metatarsal organs, and genetic mouse models, researchers have discovered that the proliferation of chondrocytes is inhibited by FGFR3 signaling.^41^, ^42^, ^43^, ^44^ The incorporation of [3H]thymidine labeling in the epiphyseal chondrocytes of cultured fetal rat metatarsals was reduced by FGF2 treatment,^45^ while severely reduced proliferation was found in the chondrocyte cell line CFK2 expressing the mutant FGFR3 that causes ACH. These findings indicate that activated FGFR3 impairs the proliferation of chondrocytes.^46^ ACH mice exhibit elevated levels of P16 and P19 proteins in their growth plates.^39^ In the epiphyseal cartilage of achondroplastic children with G380R mutation or some other mutations in FGFR3, the level of p21 expression is increased. Administration of FGF2 can induce the up-regulation of P21^WAF1^ and p27^Kip1^, cell cycle inhibitors, and inhibit the proliferation in rat chondrosarcoma cells.^47^^,^^48^ FGF2 treatment caused an elevation in the expression of p21, leading to the formation of complexes involving cyclin D2-Cdk4-p21, cyclin D2-Cdk2-p21, and cyclin E-Cdk2-p21, and reducing the activity of cyclin-dependent kinase 2 (Cdk2) and cyclin E-dependent kinases in rat chondrosarcoma cells.^49^ The data mentioned above indicate that the suppression of the proliferation of chondrocytes in the growth plate, caused by activated FGFR3, could be due to the increased expression of cell-cycle inhibitors. Moreover, FGFR3 hinders chondrocyte proliferation by diminishing telomerase activity through the down-regulation of telomerase reverse transcriptase expression.^50^ FGFR3 also regulates chondrocyte proliferation by interacting with the Indian hedgehog (IHH)/PTHrP signaling pathway, which is crucial for the transformation of chondrocytes from the stage of proliferation to hypertrophy.^51^ In mouse models of FGFR3-related dysplasia, FGFR3 suppresses IHH signaling in both growth plates and perichondrium.^52^, ^53^, ^54^ The activation of FGFR3 has been found to suppress PTH/PTHrP signaling via the JAK (Janus kinase)/STAT (signal transducer and activator of transcription) pathway, whereas the restoration of PTHrP signaling can counteract the negative impact of FGFR3 on chondrocyte proliferation.^55^^,^^56^

The role of FGFR3 in chondrocyte differentiation remains a subject of controversy. Lacking Fgfr3 results in an elevation in bone length along with enhanced hypertrophy of chondrocytes in mice.^34^^,^^57^ Chen et al revealed that ACH mutation resulted in a decrease in hypertrophic zone height in the growth plates. Conversely, Minina and colleagues discovered that administration of FGF2 in limb explant culture can expedite the hypertrophic differentiation of chondrocytes.^39^^,^^58^ Previous studies have demonstrated that the addition of FGFs (FGF1/2/7) can boost the expression of Sox9 in mouse primary chondrocytes and C3H10T1/2 cells. Furthermore, the increased expression of Sox9 induced by FGFs in mesenchymal and prechondrocytic cells is mediated by the MAPK (mitogen-activated protein kinase) pathway.^59^ These findings support the hypothesis that an excessively high expression of Sox9 may contribute to the development of ACH.^60^ Additional research using a mouse model has revealed that a crucial mechanism contributing to the impaired growth of endochondral bone in disorders resulting from FGFR3 mutations is a blockage in chondrogenic differentiation, which is not regulated by cell division but relies on Sox9,^61^^,^^62^ particularly in the joint formation and growth plate development. FGF9/18, a high-affinity ligand of FGFR3, can stimulate early chondrogenic differentiation and delay terminal hypertrophy during human mesenchymal stem cell-dependent chondrogenesis.^63^ Mice lacking Fgf9 display rhizomelia, which is comparable to chondrodysplasia syndromes caused by activated FGFR3, and it has been revealed that FGF9 promotes hypertrophy of chondrocytes at early stages and regulates vascularization and osteogenesis at later stages in developing stylopod elements.^64^ Conversely, other studies have demonstrated that FGFs/FGFR3 signaling promotes the terminal hypertrophic differentiation of chondrocytes, partly via the MAPK pathway.^58^^,^^65^

The involvement of FGFR3 in programmed cell death is still a subject of controversy due to contradictory results. Several studies indicate that FGFR3 promotes apoptosis in chondrogenic ATDC5 cells via caspase activity, partially through the PLCγ (phospholipase C gamma)-STAT1 pathway, and suppresses the expression of PTHrP and Bcl-2.^66^^,^^67^ On the other hand, Henderson et al discovered that FGFR3 G380R mutation inhibited the serum deprivation-induced cell death in CFK2 cells.^46^ The extracellular signal-regulated kinase (ERK) pathway is essential for reversible premature senescence, characterized by growth arrest in the rat chondrosarcoma cell line, triggered by the activation of intrinsic FGFR3.^68^

The FGFR3 signaling pathway has been found to have a negative impact on the extracellular matrix of chondrocytes. This is accomplished by suppressing the synthesis of the extracellular matrix of chondrocytes, specifically the production of ACAN and Col2,^47^^,^^69^ while also facilitating extracellular matrix degradation. The degradation process is facilitated by the stimulation of several MMPs, including MMP3/9/10/13.^70^ Additionally, signaling pathways regulating the downstream molecules of FGFR3, such as Snail and C-type natriuretic peptide (CNP) signaling, have been identified in chondrocytes.^71^, ^72^, ^73^ Additionally, the activated mutations in FGFR3 have been demonstrated to exert a vital role in causing growth plate defects due to dysfunction in primary cilia. The ACH-related dysfunction of primary cilia may manifest as a decrease in length and reduced trafficking of intraflagellar transport 20 (IFT20) or impaired IHH signaling.^74^^,^^75^ These data provide new insights for the development of therapeutic agents for ACH.

### FGFR3 signaling in osteogenesis process

The long bone of mice mimicking human ACH displays increased levels of various markers associated with osteogenic differentiation,^39^ indicating the involvement of FGFR3 in the regulation of osteogenic differentiation. Moreover, cranial vault defects are caused by gain- or loss-of-function mutations in FGFR3, which highlights the important role of FGFR3 in intramembranous ossification.^76^ Mice with FGFR3 P244R mutation, which causes human Muenke craniosynostosis syndrome, exhibit a reduction in the thickness of cortical bone and lower bone mineral density in long bone.^77^ According to our study, the presence of ACH mutation in FGFR3 hinders the proliferation of stromal cells in bone marrow and enhances their ability of osteogenic differentiation.^78^ Duplan's study discovered that the activation of FGFR3 in hypertrophic chondrocytes and immature osteoblasts (Osx-Fgfr3) in mice not only disturbed the hypertrophic cells in the growth plate, impacting the growth of long bones but also caused osteopenia and decreased cortical thickness at adulthood. Nevertheless, the activation of FGFR3 in mature osteoblasts (Col1-Fgfr3) has a limited impact on the structure, dimensions, and micro-structure of the skeleton.^79^ These findings indicate that immature osteoblasts are affected by Fgfr3 overactivation, contributing to the bone phenotypes observed in ACH. Our work, along with other studies, has further verified the direct impact of FGFR3 on the process of bone ossification in zebrafish.^80^^,^^81^

Previous studies have also indicated that FGFR3 signaling indirectly regulates osteogenesis. Expressing activated FGFR3 mutation in Col2a1 (collagen type II alpha 1 chain) positive cells results in premature closure of synchondroses and increased osteoblastic differentiation around synchondroses by up-regulating the expression of bone morphogenetic proteins (Bmps) and down-regulation of BMP antagonist.^82^ According to Mugniery's research, the disruption of growth plates resulting from the activation of FGFR3 signaling is responsible for the abnormalities observed in the trabecular bone.^83^ This implies that dysregulated FGFR3 signaling pathways may impact the formation of trabecular bone during growth through a paracrine mechanism. Furthermore, our investigation suggests that the absence of Fgfr3 in chondrocytes enhances osteogenesis by stimulating the differentiation and mineralization of osteoblasts via increased expression of Ihh, Wnt4, Bmp2, Bmp4, Bmp7, and transforming growth factor beta 1 (Tgfb1), while simultaneously reducing the expression of Noggin 84. Either FGFR3 deficiency or constitutive activation results in osteopenia and disrupts the mineralization of bone, accompanied by altered osteoclastic activity.^78^^,^^85^

Using single-cell sequencing and cell-lineage tracing, a recent study showed that fetal chondrocytes of the cartilage template marked by Fgfr3-CreER contributed to multiple cell types, such as chondrocytes, perichondrial cells, stromal cells in the metaphyseal marrow, and cortical and trabecular osteoblasts.^86^ Another study revealed Fgfr3 positive bone marrow endosteal stromal cells as a novel subtype of skeletal stem cells, abundant in young bone marrow. These cells can differentiate into osteoblasts and chondrocytes, serving as a significant source of osteoblasts that contribute to osteogenesis.^87^ It is yet to be investigated whether FGFR3 enhancements affect the functioning of skeletal stem cells associated with ACH bone phenotypes, and the precise mechanism underlying this influence necessitates further research.

### FGFR3 signaling in articular cartilage and intervertebral disc

According to reports, certain individuals with thanatophoric dysplasia display fusion of joints,^88^ and there is a high expression of *Fgfr3* in the outer interzone^89^ that gives rise to articular cartilage, suggesting that FGFR3 plays essential functional roles in joint development and homeostasis. An improved and enduring expression of the chondrogenic factor Sox9 is involved in the differentiation block and joint malformation in the mouse model of thanatophoric dysplasia type II.^61^ Despite having abnormal alignment of the lower extremity and higher rates of obesity, individuals with ACH have a lower occurrence of osteoarthritis. This is noteworthy, as osteoarthritis is typically associated with risk factors such as mechanical instability and obesity.^90^ This manifestation implies that the ACH mutation might confer a protective effect against osteoarthritis. By utilizing mouse models of osteoarthritis, in which FGFR3 is selectively deleted or activated, we have demonstrated that FGFR3 plays an essential role in delaying the progression of osteoarthritis in temporomandibular and knee joints, partially by suppressing IHH signaling in articular chondrocytes.^91^^,^^92^ Moreover, our investigation uncovers that the lack of FGFR3 in macrophages contributes to the development of synovitis and leads to the destruction of multiple joints in mice. FGFR3 inhibits the expression of C-X-C chemokine receptor type 7 (CXCR7) and therefore the chemotaxis of macrophages in response to C-X-C motif chemokine ligand 12 (CXCL12) by modulating the NF-κB (nuclear factor kappa B) pathways.^93^ In addition, FGFR3 participates in the regulation of other signaling associated with osteoarthritis. For instance, the decrease in FGFR3 expression is involved in the initiation of osteoarthritis induced by mTORC1 (mechanistic target of rapamycin complex 1) activation.^94^

Symptoms of lumbar stenosis in individuals with ACH are probably due to central canal stenosis resulting from degenerative disc disease, rather than genuine foraminal stenosis.^95^ Notably, typical changes in the intervertebral disc have been observed in a mouse model mimicking ACH, encompassing a reduction in the size of the endplate and disproportional sizes of the annulus fibrosus and nucleus pulposus.^96^ FGFR3 is exhibited in both fetal and degenerated human nucleus pulposus tissue,^97^^,^^98^ suggesting the involvement of FGFR3 in intervertebral disc development and homeostasis. During postnatal growth, it was noticed in a fate-mapping study that nucleus pulposus cells expressing FGFR3 (FGFR3 positive) experienced proliferation from the outer to inner areas of the nucleus pulposus. Through the utilization of Confetti mice for clonal lineage tracing and the diphtheria toxin A (DTA) approach for the ablation of FGFR3-expressing nucleus pulposus cells, we discovered that the morphogenesis and homeostasis of the postnatal nucleus pulposus tissue relied on the expansion of FGFR3 positive cells. Furthermore, in a mouse model of intervertebral disc degeneration and regeneration, FGFR3 positive nucleus pulposus cells exhibited significant expansion during the recovery phase.^99^ A recent study has revealed the up-regulation of FGF2 in the highly degenerative endplate. Furthermore, the effect of FGF2 transitions from facilitating anabolic processes in the early stages to accelerating catabolic mechanisms as degeneration progresses in the endplate,^100^ suggests that FGFR3 signaling might have a crucial function in intervertebral disc homeostasis.

## Approaches to treating ACH

**Figure 2:**
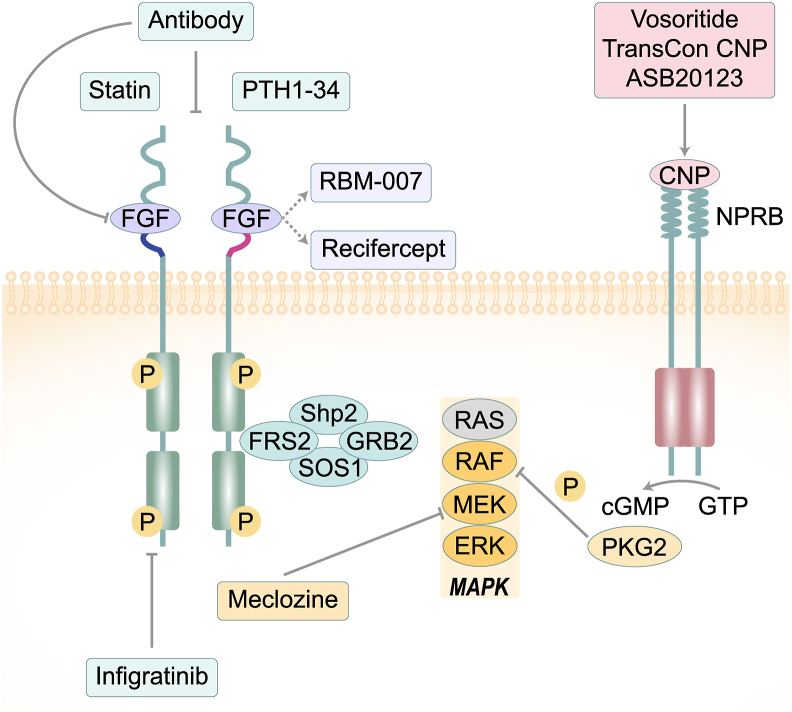
Treatment strategies for achondroplasia. FGF ligands initiate the activation of FGFR3 through the process of dimerization and transphosphorylation (P) in the intracellular kinase domain. When FGFR3 is activated, it recruits adaptor molecules (FRS2, SHP2, GRB2) that facilitate the recruitment of guanine nucleotide exchange factor SOS1 to RAS, leading to the activation of the RAS/ERK signaling module. The current therapeutic drugs under clinical evaluation for achondroplasia aim to down-regulate FGFR3 signaling through various mechanisms, such as neutralizing FGF ligands (recifercept, RBM-007), inhibiting FGFR3 catalytic activity (infigratinib), or directly inhibiting the RAS-ERK pathway (meclozine). On the other hand, TransCon CNP and vosoritide function as stable ligands within CNP pathway, effectively impeding the FGFR3 pathway through the facilitation of PKG2-mediated inhibitory phosphorylation of RAF. The potential of anti-FGFR3 antibodies lies in their ability to hinder the interaction between FGF and its receptor site or prevent FGFR3 dimer formation, whereas statins facilitate the degradation of FGFR3. FGFR3, fibroblast growth factor receptor 3; FGF, fibroblast growth factor; FRS2, fibroblast growth factor receptor substrate 2; SHP2, Src homology region 2-containing protein tyrosine phosphatase 2; GRB2, growth factor receptor bound protein 2; SOS1, son of sevenless homolog 1; CNP, C-type natriuretic peptide; PKG2, protein kinase G2; cGMP, cyclic guanosine monophosphate; PTH, parathyroid hormone; NPRB, natriuretic peptide receptor type B; MAPK, mitogen-activated protein kinase.

The complex involvement of FGFR3 in skeleton development and the discovery of the underlying mechanisms of ACH have helped the development of complex surgical procedures and identification of the potential targets for biological treatments. Here, we will introduce the current and potential future therapeutics of ACH (as shown in Fig. 2).

### Surgical approaches

In cases of both proportional and disproportional dwarfism, the use of extended limb lengthening is suggested as a feasible choice for increasing height. During surgical limb lengthening, the cortical long bones are cut through the osteotomy, and external fixators are subsequently positioned both proximal and distal to the osteotomy site. Gradual distraction is then applied over several months to achieve bone lengthening.^29^ Following the implementation of various procedures on the femurs and tibias, ACH patients have the potential to attain a maximal 25 cm increase in adult height.^101^ However, despite the partial improvement in functionality and quality of life achieved through surgical intervention, this challenging procedure is still associated with various complications, including infection, muscle contractions, and an increased risk of fracture. A modified intramedullary fixation system may improve the outcome with lower pain and risk.^102^ Assessing the substantial risk of complications compared with the potential improvement of short height presents a significant challenge when deciding before surgery. Furthermore, the surgical alleviation of the malformed spine and skull in individuals with ACH presents considerable difficulty. In forthcoming times, the integration of surgical limb lengthening alongside pharmacological interventions holds promise for further augmenting outcomes.

### Therapies with growth hormone

Recombinant human growth hormone (rhGH) therapy trials have been performed on ACH subjects for almost 50 years. Multiple studies have presented evidence supporting that short-term rhGH therapy exhibits greater efficacy in enhancing growth velocity compared with long-term treatment. It is worth noting that the most substantial increase in height may be observed during the first year of medicine administration.^103^^,^^104^

The administration of rhGH over about 10 years led to an average height increase of 3.5 cm for males and 2.8 cm for females. The combined administration of l-thyroxine and rhGH led to a definitive growth enhancement of 10.0 cm in males and 9.8 cm in females. When this therapy was combined with surgical tibial and/or femoral elongation, the ultimate height of males increased by 17.2 cm and that of females by 17.3 cm, respectively.^105^ A meta-analysis of rhGH treatment for ACH utilizing a large patient population revealed that data regarding body disproportion in rhGH therapy is unclear.^104^

### Therapies targeted at FGFR3 signaling

Numerous non-surgical approaches have been suggested to promote the longitudinal bone growth of ACH mice by reducing the activity of FGFR3, which is the primary cause of ACH due to its excessive activation. Given the shared mutations between skeletal dysplasia associated with FGFR3 and FGFR3-dependent cancers like urothelial carcinomas, cervical carcinomas, and multiple myeloma, some therapeutic strategies of ACH have been co-opted conceptually from the oncological field. There are several strategies for targeting FGFR3 protein, including soluble decoy receptors or monoclonal antibodies. Decoy receptors against FGFR3 (soluble FGFR3, sFGFR3) containing mainly FGFR3 extracellular domain have been proven to compete with FGFR3 binding to endogenous FGF ligands, leading to decreased mortality and improved skeletal growth of ACH mice through functionally regulating chondrogenesis.^106^ As a decoy receptor, recifercept made by Pfizer (formerly Therachon) prevents mutant FGFR3 from binding to FGFs.^107^ The study unveiled that recifercept could promote the growth of long bone and reverse the decreased body weight in transgenic ACH mice. Currently, it is undergoing a phase II trial for ACH participants (NCT05116046) as part of an open-label extension. Through phage display, we discovered that a peptide with inhibitory properties against FGFR3 was able to partially alleviate the growth retardation resulting from the gain-of-function mutation of FGFR3.^108^ FGFR3-specific monoclonal antibodies, vofatamab, showed great effectiveness in inhibiting the progression of FGFR3-associated bladder cancer,^109^ which provides the potential for the usage of FGFR3 antibodies in treating ACH.

The substitution of G380R, located in the transmembrane domain of FGFR3, which is responsible for most of ACH cases, has increased both ligand-dependent and ligand-independent activation of downstream signaling pathways. Therefore, FGF ligand traps offer a feasible choice for treating ACH. RBM-007 is isolated as an inhibitory RNA aptamer against the FGFR3 ligand FGF2, which has been used in a clinical trial study for exudative age-related macular degeneration.^110^ RBM-007 effectively mitigated the proliferation arrest, premature senescence, disrupted hypertrophic differentiation, and degradation of cartilaginous extracellular matrix, caused by FGF2/FGFR3 signaling in cultured chondrocytes or embryonal tibia explant. The use of RBM-007 improved the differentiation and maturation of chondrocytes in cartilage xenografts derived from induced pluripotent stem cells of ACH individuals. Notably, RBM-007 successfully reinstated long bone growth and restored growth plate morphology in ACH mice.^111^

Small molecules, FGFR-selective tyrosine kinase inhibitors that reduce mutant FGFR3-induced enhanced tyrosine kinase activity have been studied extensively. Various tyrosine kinase inhibitors have been assessed in experimental models that mimic ACH. A31, a compound that inhibits the continuous phosphorylation of FGFR3 caused by gain-of-function mutation of FGFR3, in particular, demonstrated the ability to restore the size of embryonic femurs through a culture system *ex vivo* by alleviating the expression of cell cycle regulators and promoting the hypertrophic differentiation of pre-hypertrophic chondrocytes.^112^ In ACH-like mice, the administration of NVP-BGJ398, a highly selective tyrosine kinase inhibitor targeting FGFR3,^113^ was found to effectively reduce the activation of FGFR3 (p.Tyr367Cys) and improve skeletal maldevelopment *in vivo*.^96^ The safety and efficacy of this tyrosine kinase inhibitor, known as infigratinib and developed by QED therapeutics, is currently being investigated through the PROPEL and PROPEL 2 studies. The PROPEL study (NCT04035811) aims to observe the natural progression and collect baseline data of children with ACH over 6−24 months, followed by the PROPEL 2 study (NCT04265651). In the PROPEL 2 study, children aged 3−11 years with ACH who have completed over 6 months in the PROPEL study will receive treatment with infigratinib.^114^ ASP5878, a cancer-fighting medication that specifically targets FGFRs, could elongate the femurs of a male ACH mouse model. Despite being less efficient in promoting bone elongation compared with the CNP analogue, ASP5878 offers the advantage of oral administration.^115^ Based on the safety and pharmacokinetic studies, it is possible that these small molecules could be suitable for further assessment in clinical trials involving individuals with ACH.

Advances in stem cell and gene editing technologies offer potential tools and strategies for the study and treatment of ACH. By utilizing CRISPR-Cas9 to precisely correct the mutant FGFR3, the chondrogenic differentiation of induced pluripotent stem cells derived from ACH patients was successfully restored.^116^

### Potential therapies targeted at FGFR3 downstream signaling

By inhibiting overactive FGFR3 signaling, meclozine, an antagonist of histamine H1 receptors, has been found to enhance the proliferation and differentiation of chondrocytes through down-regulation of ERK phosphorylation.^117^ Administering meclozine orally to mice with ACH resulted in longitudinal bone growth enhancement and was safe in children with ACH, with no occurrence of serious side effects.^118^^,^^119^

CNP serves as an endogenous ligand that triggers endochondral ossification via a pathway alternatively mediated by cyclic guanosine monophosphate.^120^ The stimulation of cyclic guanosine monophosphate impedes the activation of MAPK, thereby partially counteracting the inhibitory effects of FGFR3 on endochondral bone formation.^121^ ASB20123, a chimeric peptide consisting of CNP (1−22) and human ghrelin (12−28, E17D), shows potential as a therapeutic intervention for individuals with short statures.^122^ BMN111/vosoritide, a biological analog of CNP with a prolonged half-life in the bloodstream, has been developed by BioMarin Pharmaceutical. The phase 3 extension study of BMN111 has substantiated that the improvement in growth velocity remains sustained for a duration of up to 2 years, resulting in a notable enhancement in body proportions.^123^ TransCon CNP (Ascendis Pharma) is another CNP derivative, which is a novel prodrug designed to provide continuous CNP release at the growth plate, offering the possibility of once-weekly therapy. In a phase 2 clinical trial involving children aged 2–10 years diagnosed with ACH, the administration of TransCon CNP demonstrated safety, efficacy, and minimal occurrence of injection site reactions. It may offer a new, once-weekly treatment choice for kids with ACH.^124^

The identification of novel and/or relevant substances that target the FGFR3 signaling pathway has important implications for the therapeutic management of ACH. Martin et al conducted a study wherein they discovered that (−)-epicatechin, a phenolic compound derived from *Theobroma cacao*, obstructed the downstream pathways of FGFR3 effectively. Furthermore, (−)-epicatechin exhibited the capacity to improve bone elongation and rectify primary cilium abnormalities observed in chondrocytes of mice with ACH. These findings suggest that (−)-epicatechin exhibits promise as a pharmaceutical agent for the treatment of ACH.^125^

### Targeting non-FGF signaling pathways

In the growth plate of ACH, there is a significant disruption in the balance between the proliferation and differentiation of chondrocytes. The IHH/PTHrP pathway is widely recognized as the primary regulator of chondrocyte proliferation and differentiation within growth plates.^126^ Our research demonstrates that intermittent systemic PTH (1−34) (teriparatide) injections partially ameliorated the delayed skeletal development observed in ACH mice. Adding statins to the culture media effectively restored the impaired chondrogenesis observed in chondrocytes derived from induced pluripotent stem cells generated from cells of individuals with ACH. Furthermore, *in vivo* experiments demonstrated an amelioration in the skeletal phenotype of ACH mice after statin treatment.^127^ Nevertheless, the utilization of statins for the treatment of ACH remains a subject of controversy, as Fafilek et al have concluded that the FGFR signaling pathway is not inhibited by statins in chondrocytes.^128^

## Conclusion and perspective

In the past decades, advancements have been achieved in understanding the fundamental roles of FGFR3 signaling in regulating skeletal development and homeostasis, and in developing effective therapeutic strategies for FGFR3-associated skeleton dysplasia. However, several concerns need to be considered to deepen our understanding of the mechanisms of ACH and improve its treatment.

The predominant focus of research lies in the pursuit of methods to augment the elongation of ACH bones, while insufficient attention is given to fortifying the atypical bone mass, skull, and vertebrae of ACH. Furthermore, most therapeutic medications for ACH primarily focus on inhibiting MAPK, with limited investigation conducted on the regulation of alternative downstream signals of FGFR3, such as protein kinase B (AKT), and the corresponding interventions.

In the era of a variety of advanced techniques coming forth, we have great opportunities to advance our understanding the mechanisms of ACH and find more efficient treatments with less side effects. For example, with the help of single-cell analysis, it is crucial and possible to thoroughly examine the specific subset of cells regulated by FGFR3. FGFR3 signaling encompasses multiple classical downstream signaling pathways, such as MAPK, PI3K (phosphoinositide 3 kinase)/AKT, and PLCγ. Each downstream pathway has a distinct role in the development, maintenance, and diseases of the skeleton. Through the implementation of an allelic series of knock-in point mutations designed to disrupt the downstream pathways of Fgfr1 individually and in combination, Brewer et al discovered that Erk1/2-independent signaling pathways functioned importantly in mediating FGF signaling *in vivo*.^129^ Gain-of-function mutations in FGFR3 cause ACH by dysregulating multiple processes of endochondral ossification via respective downstream pathways. Among them, activated AKT is responsible for the inhibited chondrocyte proliferation, while MAPK activation exerts an inhibitory effect on matrix synthesis of FGFR3.^130^ With our increasing understanding of the functions of particular amino acids or domains in mediating distinct downstream signaling pathways, there may arise chances to selectively regulate these specific downstream pathways of FGFs/FGFR3 signaling to achieve more optimal outcomes in alleviating disease phenotypes.

Additionally, FGF signaling in intranuclear and endocrine components of skeleton development requires further investigation. For example, bent bone dysplasia partly results from a reduction of FGFR2 signaling at the plasma membrane accompanied by mutant FGFR2 protein restricted to the nucleus.^131^ These distinct functions of FGFR2 in the cell membrane and nucleus will provide new insight into FGF signaling. Furthermore, FGF23, as an endocrine FGF, which mainly binds with FGFR1c, FGFR3, and FGFR4 may have a direct inhibitory effect on bone mineralization mediated by FGFR3 in osteocytes.^132^ Additional investigation is required regarding the post-transcriptional alteration of individual FGF and FGFR during skeletal development and diseases. Emerging evidence suggests that FGFs/FGFRs signaling plays a vital role in various cellular processes essential for the maintenance of cartilage, including autophagy,^73^ senescence, and programmed cell death both in normal and pathological conditions. Nonetheless, the regulation of these processes in skeletal development and diseases by FGF signals remains uncertain and requires further investigation. The metabolic regulation of different types of skeletal cells is influenced both by their functions and their microenvironments.^133^ Whether mutant FGFR3 leads to metabolic dysregulation of distinct skeletal cells in ACH needs to be reflected in the coming years.

The complicated interactions among individual FGFs and FGFRs have been widely acknowledged. It is commonly understood that individual FGFs can simultaneously bind to FGFRs with varying affinities. However, there is a lack of comprehensive investigations into the specific mechanisms by which each FGF activates its respective FGFR. By selectively deleting or activating individual FGFs or FGFRs within specific cell types, such as chondrocytes, we can dissect the precise contributions of each FGF or FGFR in the development and homeostasis of targeted tissues. Further investigation is necessary for the dissecting of the downstream signaling pathways of FGFR3 in the regulation of the communication between cortical/trabecular bone and growth plate in ACH development. Considering our current findings about ACH based on limited histopathological analysis, the function of subgroup cells in growth plates and their roles in the pathogenesis of ACH are not clarified. The utilization of multiomics analysis will help in elucidating the attributes and roles of different clusters of cells, identifying particular cell markers, and establishing a basis for more accurate intervention. Thus, we can improve the targeting approaches, such as aptamer or peptide-based targeted modulation of FGF signaling in critical cells and tissues. Big animal models should be helpful for the mechanistic investigation and preclinical studies about the therapeutic effects of surgical methods and innovative drugs.

The FGFs/FGFR3 pathway has extensive crosstalk with other signaling during the skeleton development as well as disease processes such as ACH and cartilage degeneration. Elucidating the interplays between FGF signaling and other pathways, such as BMP, Wnt, retinoid, and hedgehog pathways, will offer valuable insights into the underlying molecular mechanisms and exploring the comprehensive and holistic treatments for the growth plate maldevelopment and other phenotypes of ACH. Recent studies have substantially enhanced our comprehension of the mechanisms underlying ACH pathogenesis, and therefore have developed several potential clinically effective biological therapies for ACH patients. With the fast development of advanced biological and medical theories and techniques, we believe before long we will have a more thorough understanding of ACH mechanisms and develop more effective biological therapies for ACH.

The FGFR3 signaling pathway emerges as a pivotal negative regulatory machinery of cartilage development. Given that, endeavors to understand this pathway come in at least two additional flavors and thus warrant future studies. First, whether the aberrant FGFR3 signaling pathway, particularly its hyperactivation, contributes to a broader spectrum of chondrodysplasias than ACH. Second, suppressing FGFR activation could be leveraged to boost cartilage growth so that many clinically well-tested FGF receptor inhibitors might be repurposed to treat common types of chondrodysplasias. Indeed, our ability to translate the molecular underpinnings of cartilage development into therapeutic approaches is still abysmal today. Using the FGFR3 gene as a springboard, we are hopefully starting to see glimpses of that in future studies.

## Funding

This work was supported by the 10.13039/501100012166National Key Research and Development Program of China (No. 2018YFA0800802), the 10.13039/501100001809National Natural Science Foundation of China (No. 82122044, 81830075), the Joint Funds of the National Natural Science Foundation of China (No.U23A20411) and the Chongqing Science Fund for Distinguished Young Scholars (No. CSTB2023NSCQ-JQX0023).

## Conflict of interests

The authors declared no conflict of interests.
